# Twitter-Based Sentiment Analysis and Topic Modeling of Social Media Posts Using Natural Language Processing, to Understand People’s Perspectives Regarding COVID-19 Booster Vaccine Shots in India: Crucial to Expanding Vaccination Coverage

**DOI:** 10.3390/vaccines10111929

**Published:** 2022-11-15

**Authors:** Praveen SV, Jose Manuel Lorenz, Rajesh Ittamalla, Kuldeep Dhama, Chiranjib Chakraborty, Daruri Venkata Srinivas Kumar, Thivyaa Mohan

**Affiliations:** 1 Department of Management Studies, National Institute of Technology, Tiruchirappalli 20015, Tamil Nadu, India; 2 Centro Tecnológico de la Carne de Galicia, Adva. Galicia n° 4, Parque Tecnológico de Galicia, San Cibrao das Vinus, 32900 Ourense, Spain; 3 Facultade de Ciencias de Ourense, Universidade de Vigo, Área de Tecnoloxía dos Alimentos, 32004 Ourense, Spain; 4 Department of Management Studies, Indian Institute of Technology, Hyderabad 502285, Telangana, India; 5 Division of Pathology, Indian Veterinary Research Institute, Izatnagar, Bareilly 243122, Uttar Pradesh, India; 6 Department of Biotechnology, School of Life Science and Biotechnology, Admas University, Kolkatta 700126, West Bengal, India; 7 School of Management Studies, University of Hyderabad, Hyderabad 500046, Telangana, India

**Keywords:** booster dose, COVID-19, NLP, topic modeling, sentiment analysis

## Abstract

This study analyzed perceptions of Indians regarding COVID-19 booster dose vaccines using natural language processing techniques, particularly, sentiment analysis and topic modeling. We analyzed tweets generated by Indian citizens for this study. In late July 2022, the Indian government hastened the process of COVID-19 booster dose vaccinations. Understanding the emotions and concerns of the citizens regarding the health policy being implemented will assist the government, health policy officials, and policymakers implement the policy efficiently so that desired results can be achieved. Seventy-six thousand nine hundred seventy-nine tweets were used for this study. The sentiment analysis study revealed that out of those 76,979 tweets, more than half (*n* = 40,719 tweets (52.8%) had negative sentiments, 24,242 tweets (31.5%) had neutral sentiments, and 12,018 tweets (15.6%) had positive sentiments. Social media posts by Indians on the COVID-19 booster doses have focused on the feelings that younger people do not need vaccines and that vaccinations are unhealthy.

## 1. Introduction

The first case of coronavirus disease (COVID-19), caused by severe acute respiratory syndrome coronavirus 2 (SARS-CoV-2), was recorded at the late end of 2020 in China, and within a few months, this novel disease rapidly spread in many countries and, consequently, led to a devastating pandemic affecting more than 200 countries worldwide [1,2,3]. At the time of writing this article—as of 5 September 2022—more than 600 million cases and nearly 6.5 million deaths have been reported worldwide, and, in India alone, there have been around 44.5 million cases and 5.28 million deaths [4]. According to a recent study published in *The Lancet*, COVID-19 vaccinations reduced the possible global death toll during the epidemic by nearly two-thirds in their first year and saved an estimated 19.8 million lives [5]. Governments worldwide have recommended that their citizens receive two doses of vaccines to gain adequate immunity [6]. While the initial two doses of COVID-19 vaccines can immunize people against severe COVID-19 cases and death, immunity tends to wane after some time, which necessitates the administration of booster shots to sustain the protective levels of immunity as SARS-CoV-2 constantly mutates and with the continuous emergence of newer variants that could evade host immunity [3,7,8]. The emerging SARS-CoV-2 variants such as Delta, Omicron, and its lineages (variants of concern, VOCs) have been found to cause significant adverse impacts by overpowering protective immunity induced by COVID-19 vaccines and antibody-based therapies, resulting in vaccine breakthrough infection, re-infection, and overall surging of cases and deaths amid different waves of the ongoing pandemic. Therefore, efforts are being made to develop more effective vaccines including variant-specific, mutation proof, universal next-generation vaccines, as well as administering more doses of vaccines (booster shots) for boosting protective immunity to safeguard health amid emerging variants [9,10,11,12,13,14,15]. Despite the development of few vaccines and the ongoing global vaccination drive, COVID-19 vaccination hesitancy, diplomacy, and inequitable access to vaccines, particularly among low- and middle-income countries, also constitute significant reasons for some hindrances in the ongoing global vaccination drive, including booster shots, which helped in the sustained global burden of COVID-19; hence, global vaccination coverage needs to be enhanced holistically [16,17,18,19,20,21].

## 2. Materials and Methods

In this study, we analyzed the social media posts of Indians to understand their perspectives regarding COVID-19 booster doses and the concerns they shared regarding booster doses. Recent studies have confirmed that one of the reliable ways to predict, control, and prevent a health crisis or pandemic is by analyzing social media data [22,23]. It is important for the government and policymakers to understand the opinions of people regarding any health policy they implement, because implementing any policy that is not supported by most of the population will lead to failure in achieving the desired results.

Since the beginning of the COVID-19 pandemic, Twitter has evolved into a medium through which people can express their experiences, emotions, and perspectives regarding health policies. Therefore, we chose tweets as the data source for our study. Several research studies were conducted during the initial days of COVID-19 using Twitter data to analyze the situation and understand the perception of common people regarding health policies and various aspects of the pandemic [24,25]. To implement a successful policy and promote adequate disease prevention strategies and public safety measures, government officials and policymakers must understand the beliefs and perceptions of citizens regarding COVID-19 vaccination and booster doses. In this study, we used natural language processing (NLP) techniques, in particular, sentiment analysis and topic modeling, to comprehend the Indian general public’s perceptions regarding the COVID-19 booster dose

### 2.1. Data Collection

Tweets with the words “COVID-19 Booster” posted by Indians after 1 March 2022 to 7 September 2022 were scraped using the Python library Twint. After we removed the tweets belonging to other languages and duplicate tweets, we were left with 76,979 English tweets. We removed the tweets that were not in English because of the nature of the tweets. The vast majority of Indian tweets in other languages (Hindi, Tamil, and Telugu) were mixed with English and a particular language. For example, most of the Hindi tweets were not written in Hindi alphabets and were written in English alphabets (Hindi words being written with the English alphabet), and, due to this, it is not possible to extract sentiment or topics out of it, and therefore we removed such tweets from our corpus. Since the tweets in our dataset were from different states in India, our results are applicable to the entire Indian population. Twint is an advanced Python Twitter scraping tool that allows researchers to access Twitter data without the need for an Application Programming Interface (API); therefore, we used it to collect data [26].

### 2.2. Data Cleaning

Data cleaning is a vital task in text analytics studies to achieve the desired results. The data cleaning process includes removing all the entities that are not needed for textual data analysis; before we started our analysis, we cleaned the data. We removed stop words, punctuations, URLs, and other unwanted entities that are not needed for the text analytics. Stop words are words such as ‘a’, ‘a’, an’, ‘an’, and ‘the’ that do not have any meaning on their own and are, therefore, not needed for the analysis. We also stemmed and lemmatized the data in our corpus. Stemming is the process of reducing words into their root type by chopping off end letters such as ‘goals -> goal’ and ‘pens -> pen’ [27]. Lemmatizing is the process in which words of a similar tree are grouped together and analyzed so that analysis can be qualitative [28].

### 2.3. Sentiment Analysis

Sentiment analysis is an automatic method for extracting and analyzing subjective judgments on different aspects of an item or entity. Sentiment analysis helps us understand the premises of the text and the emotions exhibited by the author of the text [29]. Understanding common people’s sentiments regarding a particular aspect, such as a particular health policy, can help governments and policymakers understand whether the policy they implement attracts common people. In our study, we used sentiment analysis to understand Indian social media users’ perceptions of COVID-19 booster dose vaccines. We used the Python library TextBlob for the process of sentiment analysis. The TextBlob library uses natural language processing and advanced machine learning principles to analyze every word in the documents presented in the corpus, defining the overall sentiments being projected as positive, negative, or neutral. The TextBlob library works on the bag-of-words model and a predefined dictionary classifying negative and positive words. The TextBlob library goes through each word in the document and assigns an individual score to all words, and the final score of that document is determined by a pooling operation (taking an average of all sentiments) [30].

### 2.4. Topic Modeling

**Figure 1:**
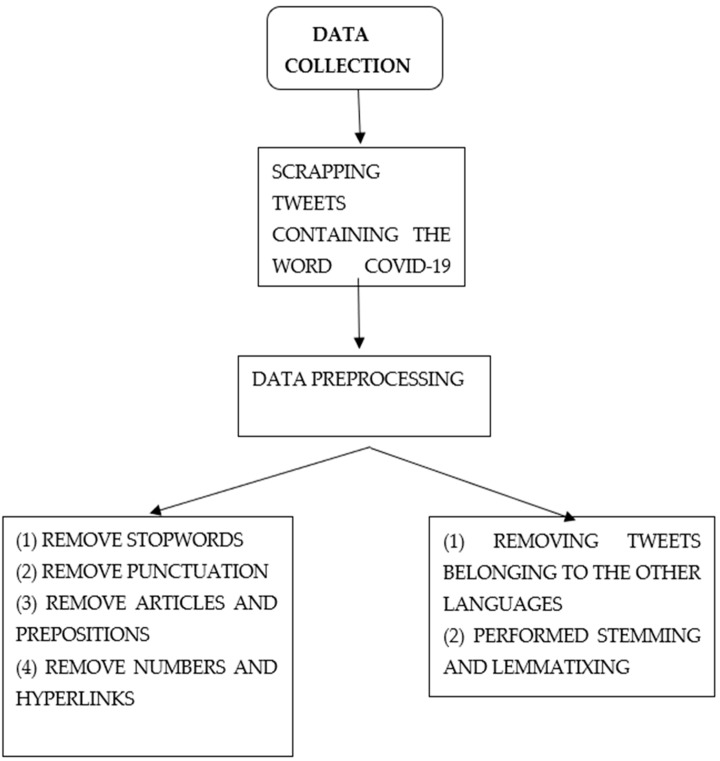
Data collection and data pre-processing.

Sentiment analysis helps us to understand the perceptions of common people regarding a particular health policy. However, the factors that drive emotions can only be understood through topic modeling. Topic modeling is a generative statistical model that captures the essence of a text. Latent Dirichlet allocation (LDA) topic modeling is a prominent technique used to understand the premises of a text, upon which the entire corpus is built. LDA algorithms follow the bag-of-words model and operate under two assumptions [31]. The LDA algorithm assumes that all documents present in the corpus are a mixture of topics, where each topic is a probability distribution over words [32]. The Dirichlet process is a probability distribution, whose range is a set of probability distributions. A graphical representation of the LDA model is shown in Figure 1.

**Figure 2:**
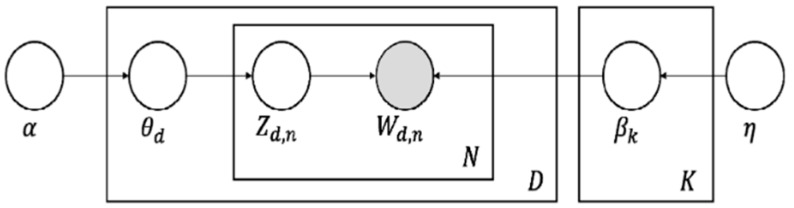
Graphical representation of LDA model.

Figure 2 provides the graphical representation of the LDA Model. All nodes in the model are random variables, and the observed variable (*Wd*,*n*) is shaded. Alpha (α) is a Dirichlet parameter. *θd* denotes the per-document topic proportion. *Zd*,*n* refers to the per-word topic assignment. *Wd*,*n* is the observed words. K refers to the different topics. N refers to the number of words in the document. *Βk* refers to the probability distribution over the top different words for a given topic K. D refers to the total number of documents. Eta (η) is a topic hyperparameter.

LDA algorithms find the latent (hidden) subjects and topics in the corpus, and the observed variables are words. The hyperparameters are alpha (α) and Eta (η). The higher the value of alpha, the higher the probability of all topics appearing, which results in skewed results. For this reason, in our model, we set the value for alpha as low as possible, as a lower alpha corresponds to the model preferring one topic with a higher probability than the other. We ran the model multiple times using different parameters to achieve the desired results. The distribution of LDA algorithms used to draw the per-document topic proportion (*θd*) is a Dirichlet distribution. The Dirichlet distribution is an exponential family distribution over the simplex (all positive vectors sum to one).

The values of *θd*, *βk*, and *Zd*,*n* were determined by computing the posterior distribution of all the parameters given the observation. The posterior distribution is a distribution of a set of unknown parameters or latent variables conditioned on the current data. For estimating the posterior probability of these parameters, the LDA model follows the Gibbs sampling method to define the posterior probability for the parameters. Gibbs sampling is a form of Markov chain Monte Carlo that practically stimulates a high-dimensional distribution by sampling on a lower dimensional subset of variables, where each subset is conditioned on the value of others. The sampling process is performed sequentially and continues until the sample values approximate the target distribution. The LDA model is used to estimate the posterior distribution over Z directly and, using the distribution, estimates of beta and theta were drawn.

Compared to previous methods such as manual content analysis and the word frequency method, LDA topic modeling is the best fit for understanding the topics based on which the corpus is built, particularly when dealing with unstructured data. Manual content analysis was the first attempt to understand the determinants of perception in textual data [33]. However, one of the significant drawbacks of manual content analysis is that the entire process relies heavily on the expertise of the expert; therefore, the results are unreliable. Next to manual content analysis, the word frequency model was used to understand the determinants of perceptions in the textual data. The major drawback of this method, however, is that the word frequency analysis method does not consider the word’s context and is merely a representation of the word counts; therefore, the conclusions based on this can often be confusing and ambiguous [34]. LDA is a standard method used by many researchers because it employs a probabilistic framework to determine and detect hidden themes and topics in the corpus by following the bag-of-words approach; therefore, we employed LDA algorithms to understand the concerns Indian citizens discuss regarding COVID-19 booster doses.

## 3. Results

This study was conducted in two parts. First, sentiment analysis was performed to understand people’s sentiments towards booster doses of COVID-19 vaccines. Sentiment analysis detects sentiments expressed by a person in a text. TextBlob algorithms examine each word in the tweet and determine whether the general sentiment of the particular text in the corpus is positive, negative, or neutral [35]. Second, LDA topic modeling was utilized to identify the major aspects that Indian social media users discussed regarding COVID-19 booster doses on social media. Topic Modeling is an assemblage of algorithms that summarizes a massive corpus of texts by independently identifying obscure subjects and themes covered by a collection of corpora. LDA adheres to the Bayesian principle, where the algorithm considers that each text in the corpus is composed of a variety of discrete topics, each of which has a multinomial word-frequency distribution [36,37,38]. A total of 76,979 tweets were used in this study. We selected an equal number of tweets every month in the corpus for an effective comparison. The sentiment analysis study revealed that out of 76,979 tweets, more than half of the tweets (*n* = 40,719 tweets (52.8%)) about COVID-19 booster doses had negative sentiments, 24,242 tweets (31.5%) showed neutral sentiments, and 12,018 tweets (15.6%) had positive sentiments. The monthly distribution of sentiments is presented in Table 1.

**Figure 3:**
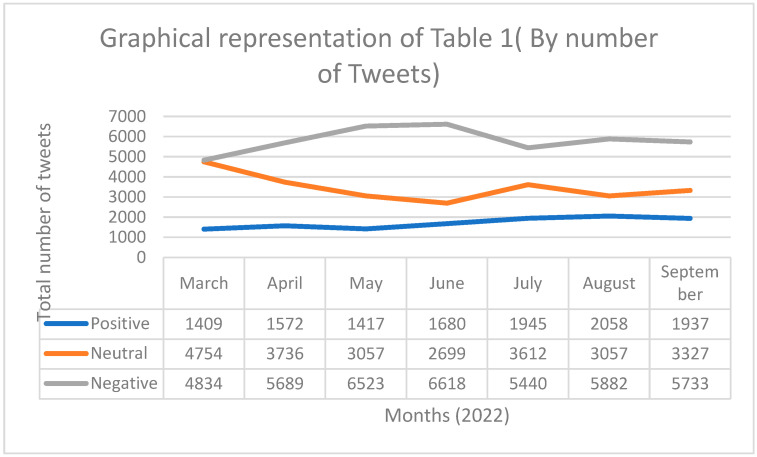
Graphical representation of Table 1 (by number of tweets).

**Figure 4:**
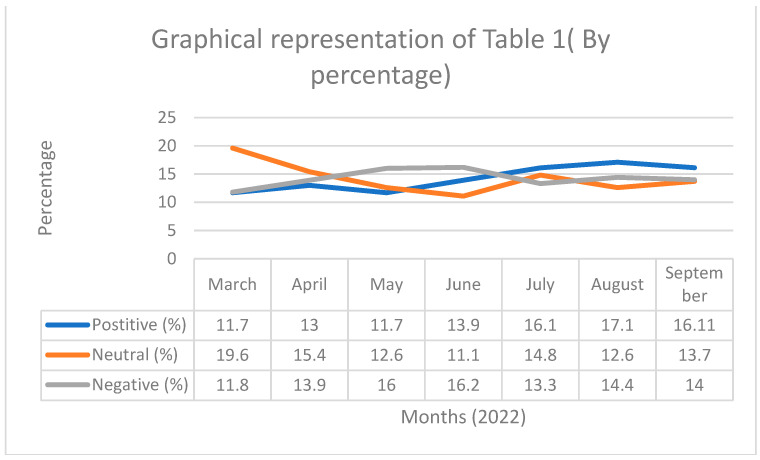
Graphical representation of Table 1 (by percentage).

Figure 3 and Figure 4 provide us the graphical representation of the Table 1. In Part 2, topic modeling was conducted on the tweets to determine the important aspects that Indians discuss when tweeting about “COVID-19 Booster Doses” on social media. For the topic modeling study, we only used tweets about COVID-19 booster doses that had negative sentiments, as the objective of the study was to understand the concerns of Indians regarding the COVID-19 booster doses. The results of topic modeling are presented in Table 2.

## 4. Discussion

Our sentiment analysis showed that nearly 84.4% of Indians‘ social media posts on the COVID-19 booster dose were either negative or neutral. A previous study analyzing Indians’ perceptions regarding the first two doses of vaccines concluded that 17% of the opinions of Indians regarding normal COVID-19 vaccines were negative and 47% of opinions regarding normal COVID-19 vaccines were neutral [39]. There was an increase of approximately 35% in the negative tone and a 16% decrease in the neutral tone. Our results show that Indians’ opinions on booster doses are more negative and polarized than the original normal COVID-19 vaccines. As shown in Figure 2 and Figure 3, the percentage of people posting about COVID-19 booster doses in a neutral sentiment fluctuated throughout the time period. Compared to March 2022, the percentage of Indians positive for booster doses increased slightly in the later months. Comparing with March, there is a considerable reduction in the percentage of people posting about booster doses in a neutral sentiment. It can be inferred from our results that when comparing to the initial months of 2022, Indians have become more polarized in their opinion regarding the booster dose vaccines.

Our topic modeling results showed that certain aspects, such as feeling that there is no need for young people to take booster doses, feeling that taking booster doses is not healthy, skepticism towards big pharma companies, fear of illness, COVID-19 vaccines not being trustworthy, feeling that normal doses of vaccines are enough, fear of severe side effects, negative perceptions created by media regarding booster doses, fear of chest pain, and feeling booster doses are unnecessary, are the concerns Indian citizens discuss about COVID-19 booster doses. With only 15.6% of the population feeling positive about booster doses, it will be difficult for Indian governments and health policymakers to encourage more citizens to take up additional vaccines. The Indian governments and policymakers should administer and promote effective awareness programs and policies through social media and all forms of necessary communications to educate the Indian public regarding the necessity of taking booster doses to achieve the desired results of protective immunity among the population and safeguard their health amid the ongoing COVID-19 pandemic under the threats of continuously emerging SARS-CoV-2 variants, sub-variants, and lineages. This research has a few limitations. We analyzed the perceptions of Indians regarding booster doses for a period of seven months. The results may vary slightly across different periods. In our research, we also did not consider the aspect of subculture that plays a role in individuals developing their perceptions of COVID-19 vaccines. Future research can analyze the aspect of subculture and how much it modifies or influences an individual’s perception of the development of perception towards COVID-19 vaccines. Further, we have only used English tweets for this study, and so our results analyzed only the perception of English-speaking people in India. Future research can focus on understanding the difference in perception of Indians speaking various languages.

## Figures and Tables

**Table 1:** Sentiment analysis.

Month	Total Tweets	Positive	%	Neutral	%	Negative	%
March 2022	10,997	1409	11.7	4754	19.6	4834	11.8
April 2022	10,997	1572	13.0	3736	15.4	5689	13.9
May 2022	10,997	1417	11.7	3057	12.6	6523	16.0
June 2022	10,997	1680	13.9	2699	11.1	6618	16.2
July 2022	10,997	1945	16.1	3612	14.8	5440	13.3
August 2022	10,997	2058	17.1	3057	12.6	5882	14.4
September 2022	10,997	1937	16.11	3327	13.7	5733	14.0
	76,979	12,018		24,242		40,719	

**Table 2:** Topic modeling.

Topic Label	Top Words
Feeling that young people don’t need booster doses Not healthy to take booster dose Skepticism towards big Pharma Fear of illness COVID-19 vaccines not trustworthy Feeling already immune enough Fear of side effects Negative perceptions created by media Chest pain Feeling not necessary	Age, dose, young, waste, booster, first Dose, booster, higher, risk, condition, health BioNTech, news, pharma, shit, profit, dose data booster, risk, COVID, severe, ill, mrna vaccines, COVID, taken, even, reinfect, distrust person, require, immune, vaccine, enough, taken pain, hand, tired, vaccine, work, high article, booster, media, news, negative, can booster, COVID, chest, pain, will, infect immune, new, healthy, food, nature, develop

Note: Top words are generated by the model. Topic names were manually created.

## Data Availability

Data are with corresponding author and will be provided upon reasonable request.
